# A Therapeutic Antiviral Antibody Inhibits the Anterograde Directed Neuron-to-Cell Spread of Herpes Simplex Virus and Protects against Ocular Disease

**DOI:** 10.3389/fmicb.2017.02115

**Published:** 2017-10-31

**Authors:** Dirk Bauer, Mira Alt, Miriam Dirks, Anna Buch, Christiane S. Heilingloh, Ulf Dittmer, Bernd Giebel, André Görgens, Vivien Palapys, Maren Kasper, Anna M. Eis-Hübinger, Beate Sodeik, Arnd Heiligenhaus, Michael Roggendorf, Adalbert Krawczyk

**Affiliations:** ^1^Department of Ophthalmology, Ophtha Lab, St. Franziskus-Hospital, Münster, Germany; ^2^Institute of Virology, University Hospital of Essen, University of Duisburg-Essen, Essen, Germany; ^3^Institute of Virology, Hannover Medical School, Hannover, Germany; ^4^Department of Immune Modulation, University Hospital Erlangen, Erlangen, Germany; ^5^Institute for Transfusion Medicine, University Hospital of Essen, University of Duisburg-Essen, Essen, Germany; ^6^Institute of Virology, University of Bonn Medical Centre, Bonn, Germany; ^7^Department of Ophthalmology, University Hospital of Essen, University of Duisburg-Essen, Essen, Germany

**Keywords:** antiviral antibody, herpes simplex virus, acute retinal necrosis

## Abstract

Herpes simplex virus (HSV) is a leading cause of blindness and viral encephalitis in the developed world. Upon reactivation from sensory neurons, HSV returns via axonal transport to peripheral tissues where it causes, e.g., severe, potentially blinding ocular diseases. In the present study we investigated whether the HSV-1/2 glycoprotein B-specific antibody mAb 2c or its humanized counterpart mAb hu2c can protect from ocular disease in a mouse model of HSV-1-induced acute retinal necrosis (ARN). In this model the viral spread from the initially infected to the contralateral eye resembles the routes taken in humans upon HSV reactivation. Systemic antibody treatment prior or early after infection effectively protected the mice from the development of ARN. These observations suggest that the antibody potently neutralized the infection and inhibited the viral transmission, since there was almost no virus detectable in the contralateral eyes and trigeminal ganglia of antibody treated mice. Besides of neutralizing free virus or limiting the infection via activating the complement or cellular effector functions, blocking of the anterograde directed neuron-to-cell spread of HSV represents a viable mode of action how mAb 2c protected the mice from ARN. We proved this hypothesis using a microfluidic chamber system. Neurons and epithelial cells were cultured in two separate compartments where the neurons sent axons via connecting microgrooves to the epithelial cells. Neurons were infected with a reporter HSV-1 strain expressing mCherry, and the co-culture was treated with neutralizing antibodies. In contrast to commercial polyclonal human HSV-neutralizing immunoglobulins, mAb 2c effectively blocked the anterograde directed neuron-to-cell transmission of the virus. Our data suggest that the humanized HSV-1/2-gB antibody protects mice from ocular disease by blocking the neuronal spread of HSV. Therefore, mAb hu2c may become a potent novel therapeutic option for severe ocular HSV infections.

## Introduction

Herpes simplex viruses (HSVs) belong to the most widespread viruses worldwide. Approximately 90% of adults are infected with HSV-1 (Bernstein et al., 2013), and roughly 16% with HSV-2 (Looker et al., 2008). HSV infection is transmitted by close body contacts, usually in early childhood from mother to child or after the onset of sexuality in intimate contacts (Anzivino et al., 2009). Primary infections are mostly asymptomatic (Koutsky et al., 1990; Langenberg et al., 1999), followed by the establishment of a life-long, latent infection in the sensory neurons of the host (Vrabec and Alford, 2004; Whitley et al., 2007). From the state of latency HSV may reactivate upon a decreased function of the immune system, resulting in asymptomatic viral shedding or recurrent clinical lesions (Balasubramaniam et al., 2014). HSV-1 and HSV-2 may cause genital, orofacial, or ocular infections, and these infections are clinically indistinguishable between HSV-1 and HSV-2 (Corey et al., 1983; Whitley and Roizman, 2001; Buxbaum et al., 2003; Lau et al., 2007; Grose, 2012; Bernstein et al., 2013). Most recurrent episodes are mild and self-limiting and can be managed by supportive antiviral therapy (Sen and Barton, 2007). However, life-threatening infections may occur when the virus invades the central nervous system, or upon disseminated disease in immunocompromised individuals (Meyding-Lamade and Strank, 2012). Serious disorders may also occur in otherwise healthy individuals, when the virus infects the eye (Lau et al., 2007; Burrel et al., 2013). Infections of the cornea may lead to the development of immune-mediated herpetic stromal keratitis (HSK) (Burrel et al., 2013), while manifestations in the retina can result in acute retinal necrosis (ARN) (Lau et al., 2007). Both clinical manifestations are hard to treat and may result in visual impairment or blindness (Lau et al., 2007; Burrel et al., 2013). HSV has evolved various means of evading or subverting normal host defenses (Zhong et al., 2013; Su et al., 2016). One of these mechanisms is the cell-associated transmission of the virus. The general term for this route of viral transmission is the “cell-to-cell spread” (Sattentau, 2008), and includes viral transmission within peripheral tissues (here, frequently called cell-to-cell spread), between neurons (neuron-to-neuron spread), between epithelial cells and neurons during primary infection (cell-to-neuron spread), and between neurons and epithelial cells after re-activation of the virus from ganglia (neuron-to-cell spread).

Recurrent HSV infections of the eye and other organs require transmission of the virus from the latently infected ganglia to the periphery via the axonal route (anterograde spread) (McGavern and Kang, 2011). Although this mechanism is not completely understood in molecular terms, it involves a *trans*-synaptic spread of HSV between neurons, the anterograde transmission from infected neurons to fibroblasts or epithelial cells, and the spread of the infection in peripheral tissues (Mikloska et al., 1999; Taylor et al., 2012a,b). The available data indicate that infectious virions are released specifically at synapses and spread across the synaptic cleft to the directly neighboring cells by fusing with the plasma membrane or with the limiting membrane of endosomes (Mikloska et al., 1999; Curanovic and Enquist, 2009). This viral spread is likely to be dependent upon the membrane glycoproteins: gB, gD, gE/gI, and gH/gL (Snyder et al., 2006; McGraw et al., 2009). Importantly, the glycoprotein B is essential to trigger a fusion process, which is essential for the viral entry into the host cell and the cell-to-cell spread between infected and non-infected cells (Heldwein et al., 2006; Sattentau, 2008). We recently described the antiviral, monoclonal antibody mAb 2c and its humanized counterpart mAb hu2c (Eis-Hubinger et al., 1993; Krawczyk et al., 2011, 2013). Both antibodies recognize a discontinuous, highly conserved epitope on the gB of HSV-1 and HSV-2 and interfere with the fusion between the viral and the target cell membranes of the host (Krawczyk et al., 2011). Furthermore, they successfully block the cell-to-cell transmission of HSV between infected and non-infected Vero cells (Krawczyk et al., 2011, 2013). Moreover, these antibodies effectively prevented mice from the development of a lethal infection in the NOD/SCID mouse model for immunosuppression (Krawczyk et al., 2013) and from HSK in the corresponding mouse model (Krawczyk et al., 2015). Besides generalized infections that may lead to lethal encephalitis and infections of the cornea resulting in HSK, infections of the cornea with HSV are a severe disease that urgently needs an improved therapy. Since HSV transmission from neurons to target cells is crucial for the spreading of the infection upon reactivation to peripheral tissues (McGavern and Kang, 2011), and gB is essential for the anterograde-directed viral spread (Curanovic and Enquist, 2009), we tested whether blocking of this route with a gB-specific neutralizing antibody could prevent mice from ocular disease.

In the present study we investigated the antiviral efficiency of this antibody and its humanized counterpart mAb hu2c in a mouse model of ARN. Similar to the emergence of ocular infection upon HSV reactivation in humans, the establishment of disease in this model requires effective viral spread from sensory neurons to the periphery (Whittum et al., 1984). Moreover, using a microfluidic chamber system (Taylor et al., 2005; Liu et al., 2008), we investigated whether mAb 2c can directly interfere with the anterograde-directed spread from neurons to epithelial cells.

## Materials and Methods

### Ethics Statement

Animal experiments were performed in strict accordance with the German regulations of the Society for Laboratory Animal Science (GV-SOLAS) and the European Health Law of the Federation of Laboratory Animal Science Associations (FELASA). The protocol was approved by the North Rhine-Westphalia State Agency for Nature, Environment, and Consumer Protection (LANUV) (Permit number: G 1194/11). Preparation of murine sensory neurons was performed according to the German Animal Welfare Act. All efforts were made to minimize suffering.

### Animals

Female BALB/c mice, 7–8 weeks of age, were purchased from Charles River Laboratories (Sulzfeld, Germany). Female C57BL/6J mice, 8 weeks of age, were purchased from Harlan Laboratories (Rossdorf, Germany) and used to isolate primary neurons. All mice were maintained under pathogen-free conditions. Experiments were performed according to the German legal requirements with the approval of the University Hospital Essen’s animal facility.

### Viruses

The HSV-1 strain KOS was propagated on Vero cells as previously described (Bauer et al., 2000). A standard plaque assay was used to determine the plaque-forming units in the virus-containing supernatants. HSV-1(17^+^)Lox-CheVP26 that has the monomeric cherry protein attached to the small capsid protein VP26 was used as a reporter virus for the infection of neuron cultures and propagated as previously described (Sandbaumhuter et al., 2013).

### Antibodies

The monoclonal antibodies mAb 2c and mAb hu2c were produced and purified as described previously (Krawczyk et al., 2011, 2013). Concentration was measured with a NanoDrop 2000 spectrometer (Thermo Scientific, Wilmington, DE, United States). Purity was confirmed by FPLC to be ≥95%. The immunoglobulin preparation Intratect^®^ (Biotest Pharma GmbH, Dreieich, Germany) was used as a source of polyclonal human HSV-neutralizing antibodies.

### Murine Model of HSV-1 Induced Acute Retinal Necrosis (ARN)

BALB/c mice at 7–8 weeks of age were anesthetized by intraperitoneal injection of ketamine hydrochloride (2 mg) and mepivacaine hydrochloride (400 ng) and infected with HSV-1 KOS by microinjection of 2.5 μl [1 × 10^5^ plaque forming unit (PFU)] of the virus preparation into the anterior eye chamber of the right eye (Berra et al., 1994; Li et al., 2008). The intraocular injections were performed with a 30-gauge needle attached to a 10 μl microsyringe (Hamilton, Reno, NV, United States). The mice were subdivided in eight groups (*n* = 10) and treated with the murine parental (mAb 2c) or the humanized (mAb hu2c) monoclonal antibody. Systemic treatment was performed by tail vein injection of 300 μg mAb 2c or mAb hu2c either at 24 h prior to infection (pre-exposure prophylaxis, P), at 24, 40, and 56 h after infection (post-exposure prophylaxis, PEP) or at day 6 post-infection (therapy, T, only mAb 2c). Topical treatment was carried out by periodical inoculations (five times a day) of the ipsilateral and contralateral eye with 5 μl (20 μg) of mAb 2c solution (in PBS; eye drops) started at 24 h post-infection until day 12. The antibody dose of 300 μg/injection was adjusted according to our prior studies where we demonstrated the efficacy of these antibodies in the NOD/SCID mouse model for immune deficiency (Krawczyk et al., 2013) and BALB/c model for HSK (Krawczyk et al., 2015). Moreover, 15 mg/kg body weight (equal to 300 μg/mouse) represents the dosage for palivizumab, the only humanized antiviral antibody (IgG1, like mAb hu2c) that was approved by the Food and Drug Administration for the reduction of serious lower respiratory tract infections caused by the respiratory syncytial virus (Brady et al., 2014). Control mice were either PBS-treated or received ACV standard therapy at 10 mg/kg body weight three times a day, until the end of the study (day 12) by intraperitoneal injection. PBS was injected three times, at 24, 40, and 56 h after infection. Mice were sacrificed 12 days post-infection. The incidence of ocular disease was determined by light microscopy using an ophthalmic surgical microscope (Zeiss, Oberkochen, Germany), and the occurrence of ARN was determined by histology of hematoxylin–eosin stained eye sections. Additionally, viral loads of the contralateral eyes were measured with a standard plaque assay. Therefore, representative mice (*n* = 6 in each group excepting the therapy group) were infected, treated as described above, and the eyes were harvested on day 8 post-infection and examined for virus. For the therapy group, the eyes were removed on day 12 of infection.

### Histology

To verify the incidence of ARN in antibody (mAb 2c/mAb hu2c) and control mice, the left eyes from these animals were isolated at day 12 after being inspected by light microscopy (as described above) and fixed using a fixing solution (64% isopropanol, 3.7% formaldehyde, 2.5% acetic acid). Subsequently, the eyes were dehydrated with isopropanol and embedded in paraffin. From the paraffin blocks 5 μm thick serial sections were cut in a mediosagittal orientation using a rotary microtome (Leica RM2135, Nussloch, Germany). For histological examination, the eye sections were stained with hematoxylin and eosin (Shandon^TM^ Instant-Hematoxylin, Thermo Fisher Scientific, Darmstadt, Germany) according to the manufacturer’s protocol. The emergence of ARN was judged by determining the occurrence of infiltrating inflammatory cells at the ganglion cell layer and the entire retina tissue. Eye sections showing tissue damage and infiltration of immune cells were classified as pathologic, whereas healthy eyes showed no infiltration of immune cells or tissue damage (Bauer et al., 2000; Grajewski et al., 2012).

### Virus Load in the Eyes

Contralateral eyes of representative mice (*n* = 6; for each group of the ARN study) were isolated on day 8 post-infection and immediately snap frozen. Subsequently, the eyes were homogenized at 4°C and serial 1:10 dilutions of the homogenate were incubated on Vero cell monolayers for 1 h at 37°C. The monolayers were covered with RPMI-agarose medium and incubated for 3 days. To determine the viral load, the cells were fixed and stained with 2% crystal violet. Plaques were counted, and the number of plaque forming unit per milliliter was calculated based on the dilution factor as previously described (Bauer et al., 2000).

### Distribution of mAb hu2c in the HSV-1 KOS Infected Eyes

To investigate whether systemically applied antibodies may reach the initially HSV-1 KOS infected eye or the retina of the contralateral eye, we examined eye tissue sections from infected mice after intravenous injection of mAb hu2c by immunofluorescence. Female BALB/c mice at 7–8 weeks of age were infected as described above. To investigate the distribution of mAb hu2c at the initially infected eye, the antibody was intravenously injected at 48 h after initial infection. To examine whether the antibody may reach the infected retina of the contralateral eye, mAb hu2c was intravenously injected at days 6, 7, or 8 after infection, the time when HSV is expected to reach the contralateral retina (Vann and Atherton, 1991). PBS was injected as a negative control. Animals were sacrificed 6 h after the injection of mAb hu2c and the eyes were harvested and immediately shock frozen in liquid nitrogen. For immunofluorescence, frozen eyes were embedded in Tissue-Tek O.C.T. medium (Sakura, Alphen aan den Rijn, Netherlands) and sectioned (7 mm) with the Frigocut 2800 microtome (Reichert-Jung, Nussloch, Germany). Sections were dried for 30 min, fixed with cooled acetone for 10 min and incubated with blocking buffer [10% fetal calf serum (FCS) in PBS] for 15 min. The ipsilateral eye sections were stained for HSV-1 infection with a mouse anti-HSV-1/2-gD-antibody (Acris Antibodies, San Diego, CA, United States) and an Alexa488-conjugated secondary anti-mouse antibody (Invitrogen, Darmstadt, Germany) and for bound mAb hu2c with a Cy3-conjugated goat anti-human IgG secondary antibody (Invitrogen, Darmstadt, Germany). All antibodies were used for staining at 5 μg/ml and diluted in blocking buffer. The contralateral eye sections were stained with a Cy3-conjugated goat anti-human IgG secondary antibody at 5 μg/ml (Invitrogen, Darmstadt, Germany) to detect mAb hu2c bound to infected cells within the retina. Additionally, eyes from day 8 post-infection were stained for HSV-1 infection with a mouse anti-HSV-1/2-gD-antibody and an Alexa488-conjugated secondary anti-mouse antibody (5 μg/ml each) to demonstrate the co-localization between HSV-1 infection and bound mAb hu2c in the retina. Nuclei were stained with Hoechst (Hoechst 33342, 1 mg/ml; Sigma–Aldrich) for 5 min according to manufacturer’s protocol. Immunofluorescence images were acquired with a Zeiss Observer Z1 fluorescence microscope at different magnifications.

### Virus Reactivation from Trigeminal Ganglia

Ipsi- or contralateral trigeminal ganglia (TG) were explanted on day 12 after infection and examined for reactivation of HSV-1 by co-cultivation with Vero cells for 4 weeks as previously described (Krawczyk et al., 2015).

### Microfluidic Chamber System

The microfluidic chamber system (Xona Microfluidics, Temecula, CA, United States) and protocols for assembling the chambers were modified from previous reports (Taylor et al., 2005; Liu et al., 2008). Briefly, glass cover slips (24 mm × 32 mm) were cleaned in an ultrasonic bath for each 10 min in ddH_2_O with dishwashing reagent, Extran AP12 (4 g/l, Merck, Darmstadt, Germany), ddH_2_O, and absolute ethanol. Dried cover slips were incubated with 80 μl of poly-L-lysine (0.01%, Sigma–Aldrich, Schnelldorf, Germany) for 10 min at 37°C and washed three times with sterile ddH_2_O. The microfluidic chambers were then placed on the cover slips and the channels were filled with 10 μl laminin solution [50–200 μg/ml in Hank’s Balanced Salt Solution (HBSS, containing 5 mM HEPES, 10 mM D-glucose, pH 7.4)]. The microfluidic chambers were then incubated at 37°C for 24 h in a humidified incubator.

### Cells

Vero cells (American Type Culture Collection, ATCC, CCL81, Rockville, MD, United States) were cultured in Dulbecco’s Modified Eagle Medium (DMEM; Life Technologies Gibco, Darmstadt, Germany) containing 10% (v/v) FCS (Life Technologies Gibco), 100 U/ml penicillin, and 0.1 mg/ml streptomycin (penicillin–streptomycin). C127I mouse mammary epithelial cells (ATCC CRL-1616) were cultured in DMEM (Life Technologies Gibco) containing 10% (v/v) FCS (PAA, Saarbrücken, Germany) and penicillin–streptomycin.

Primary cultures of dorsal root ganglion (DRG) neurons susceptible for HSV-infection were prepared as described previously (Hjerling-Leffler et al., 2007; Krawczyk et al., 2015). Briefly, 8 weeks old C57BL/6J mice were sacrificed, DRG from the cervical, thoracic, and lumbar level of the animals were dissected and collected in 1× HBSS (containing 5 mM HEPES, 10 mM D-glucose, pH 7.4). Ganglia were first digested for 20 min at 37°C with 20 mg/ml papain (Sigma–Aldrich) in a papain activation solution (0.4 mg/ml L-cysteine, 0.5 mM EDTA, 1.5 mM CaCl_2_ × 2H_2_O, pH 7.4), followed by a digestion with 10 mg/ml collagenase IV (Thermo Fischer Scientific, Waltham, MA, United States) and 12 mg/ml dispase II (Sigma–Aldrich) in 1× HBSS. Ganglia were pelleted and resuspended in 1 ml 1× HBSS and triturated using Pasteur pipettes with narrowed ends. The neuron suspension was spun for 8 min at 381 × *g* through a cushion consisting of 20% (v/v) Percoll in CO_2_-independent medium (Life Technologies Gibco) containing 10 mM D-glucose, 5 mM HEPES, 10% FCS, and penicillin–streptomycin. After removing the supernatant, the cell pellet was resuspended in 2 ml CO_2_-independent medium and finally centrifuged for 2 min at 1000 × *g*. The pellet was resuspended in 50 μl Ham’s F-12 nutrient mix medium containing 10% FCS, 50 ng/ml 2.5S nerve growth factor (NGF; Promega Corporation, Fitchburg, WI, United States) and penicillin–streptomycin. Five microliters of the resuspended neurons was plated in the somal compartment and incubated for 30 min at 37°C. The somal compartment (neuronal cell compartment) then was filled up with 200 μl and the axonal compartment (later epithelial cell compartment) with 100 μl Ham’s F-12 nutrient mix medium. The neuronal cultures were treated with 2 μM of cytosine-β-D-arabinofuranoside (Sigma–Aldrich) to eliminate any non-neuronal cells 1 day after plating. The neuron culture medium was replaced every 2 days and the neuron cultures were kept in a humidified, CO_2_-regulated 37°C incubator. After 2 weeks of cultivation, neurons were used for conducting the experiments.

### Impact of mAb 2c on the Anterograde-Directed Neuron-to-Cell Spread *in Vitro*

Primary neurons were cultured for 2 weeks in the microfluidic chambers to let the axons pass through the microgrooves from the somal to the axonal side. After 14 days, the neurons were infected by inoculation with 5 × 10^6^ PFU HSV-1(17^+^)Lox-mCherryVP26 (in 160 μl of neuronal medium) for 2 h at 37°C. The inoculation medium was removed and the neurons were washed three times with the CO_2_-independent medium before refilling the somal chambers with 300 μl of Ham’s F-12 nutrient mix medium. Before starting the infection, axonal chambers were filled with 500 μl of neuronal medium. This hydrodynamic pressure difference was assessed to prevent the unwanted diffusion of infectious virions through the microgrooves from the somal to the axonal side (Liu et al., 2008). After infection, the medium was removed from the somal and axonal compartments. C127I epithelial cells (4.4 × 10^5^ cells per chamber) were seeded on the axonal side in 500 μl of DMEM (Life Technologies Gibco) containing 10% (v/v) FCS (PAA) and penicillin–streptomycin. By these means, the cells were attached and connected to the axons. The somal chambers were then filled with 300 μl of the neuronal medium to maintain the hydrodynamic pressure. The media were additionally supplemented with the neutralizing antibody mAb 2c (150 μg/ml) or Intratect^®^ (0.1 and 1 mg/ml; Biotest Pharma GmbH). Medium without neutralizing antibodies was used as a control. After 48 h, the culture media were removed, the cell cultures washed once with PBS and fixed with 3% PFA (in PBS) for 20 min. The anterograde neuron-to-cell transmission of the virus was analyzed by fluorescence microscopy. Immunofluorescence images were acquired with a Zeiss Observer Z1 fluorescence microscope (Carl Zeiss, Oberkochen, Germany) at a 100-fold magnification.

### Statistical Analysis

**FIGURE 1:**
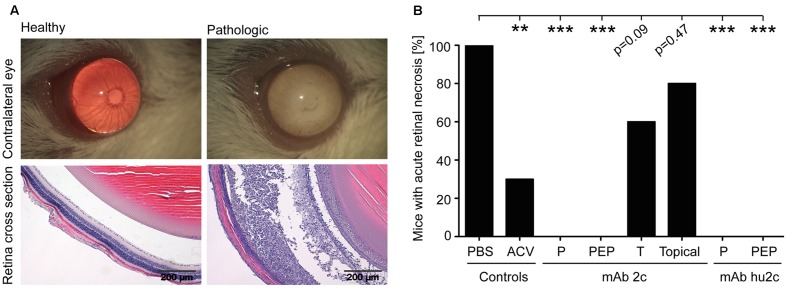
Efficacy of mAb 2c and mAb hu2c in prevention of ARN. Mice (*n* = 10) were infected with HSV-1 KOS. Antibodies were applied at 24 h prior to infection (pre-exposure prophylaxis, P), at 24, 40, and 56 h after infection (post-exposure prophylaxis, PEP) or at day 6 post-infection (therapy = T; only mAb 2c). Topical treatment with mAb 2c was started at 24 h post-infection and performed five times per day by applying antibody containing eye drops until day 12. ACV standard therapy at 10 mg/kg body weight was performed three times a day, until the end of the study (day 12) by intraperitoneal injection. The contralateral eyes were examined on day 12 post-infection for clinical signs of retinal disease by light microscopy observation and histological staining. The eyes were then classified as pathologic or healthy **(A)**. The values are given as percent of mice (*n* = 10) with pronounced retinal necrosis **(B)**. The impacts of ACV or antibody treatment were compared with the PBS group. The statistical significances were determined with the Fisher’s exact test. Comparisons were considered significant at ^∗^*P* < 0.05; ^∗∗^*P* < 0.01; and ^∗∗∗^*P* < 0.001.

**FIGURE 2:**
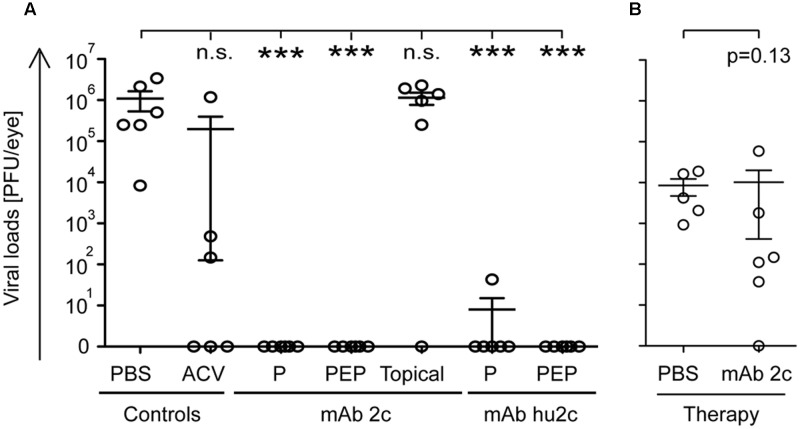
Impact of mAb 2c and mAb hu2c on the viral loads at the contralateral eye. Mice (*n* = 10) were infected with HSV-1 KOS. Antibodies were applied at 24 h prior to infection (pre-exposure prophylaxis, P), at 24, 40, and 56 h after infection (PEP) or at day 6 post-infection (therapy; only mAb 2c). Topical treatment with mAb 2c was started at 24 h post-infection and performed five times per day by applying antibody containing eye drops. ACV standard therapy at 10 mg/kg body weight was applied three times a day. The contralateral eyes were removed on day 8 **(A)** or 12 post-infection **(B)**, homogenized and inspected for viral loads using a standard plaque assay. Statistical analysis was undertaken with a non-parametric ANOVA test **(A)** or Student’s *t*-test **(B)**. Comparisons were considered significant at ^∗^*P* < 0.05; ^∗∗^*P* < 0.01; and ^∗∗∗^*P* < 0.001; n.s. = no significance. Error bars represent the SEM.

GraphPadPrism 5 (GraphPadPrism Software, La Jolla, CA, United States) was used to analyze the data. The differences between the number of mice showing signs of ARN and healthy mice (**Figure 1**) or of latently infected trigeminal ganglions and the number of trigeminal ganglions exhibiting reactivation (**Figure 5**) were examined by Fisher’s exact test. Comparisons were considered significant at ^∗^*P* < 0.05; ^∗∗^*P* < 0.01; and ^∗∗∗^*P* < 0.001. Statistical analysis of the viral loads in the eyes (**Figure 2**) was performed with non-parametric ANOVA (Kruskal–Wallis) and *post hoc* Dunn’s multiple-comparisons test. Comparisons were considered significant at ^∗^*P* < 0.05; ^∗∗^*P* < 0.01; and ^∗∗∗^*P* < 0.001.

## Results

### Efficacy of mAb 2c and hu2c in the Prevention of ARN

The vast majority of HSV-induced symptomatic diseases, such as ocular infections, emerge after reactivation of latent HSV from sensory ganglia and anterograde-directed viral spread of reactivated HSV from the ganglia to periphery (Whitley and Roizman, 2001; Whitley et al., 2007). Glycoprotein B is essential for this process (Curanovic and Enquist, 2009). Therefore, we hypothesized that the HSV-1/2 gB-targeting antiviral antibody mAb 2c and its humanized counterpart mAb hu2c might be potent candidates for the prevention and treatment of ocular HSV infections. We investigated the efficacy of mAb 2c and its humanized variant in the mouse model of ARN, since this model mimics the anterograde route of HSV transmission in humans.

To investigate whether mAb 2c or hu2c could inhibit the development of ARN, mice were infected with HSV-1 KOS and treated with mAb 2c, mAb hu2c, or ACV. At day 12 post-infection, the contralateral eyes were analyzed for typical pathological signs of an ocular HSV-1 infection. In the PBS-treated control group, all (10/10) mice developed pronounced inflammation at the anterior segment of the contralateral eye. The iris vessels were dilated and the cornea was slightly opaque. The pupils were enlarged when compared to non-infected mice. On the lens fibrin-like deposits were detected. Histological observation of the posterior part the eyes showed pronounced retinal necrosis. Exemplary pictures of healthy eyes, eyes with pronounced disease, and the corresponding histology findings are shown in **Figure 1A** and summarized in **Figure 1B**. Mice receiving ACV standard treatment (10 mg/kg; three times per day) were mostly protected from disease (**Figure 1B**). In this group, 30% (3/10) of the animals showed signs of HSV infection at the contralateral eyes (**Figure 1B**). In these animals, a pronounced retinal necrosis was detected. The severity of ARN was comparable to the PBS control group. 70% (7/10) of ACV-treated mice developed no sign for inflammation or retinal necrosis (**Figure 1B**).

It is noteworthy that the best protection from disease could be achieved when using the monoclonal antibodies. All mice systemically treated with the parental mAb 2c or the humanized mAb hu2c either at 24 h prior to infection as pre-exposure prophylaxis (P) or at 24, 40, and 56 h after infection as PEP were completely protected from the development of ARN (**Figure 1B**). Moreover, no signs of inflammation in the anterior part of the contralateral eye could be detected [P: 0% (0/10); PEP: 0% (0/10)] (**Figure 1B**). The corresponding histology examinations showed also no signs of retinal necrosis (**Figure 1A**).

To investigate the therapeutic potential of this antiviral antibody during an on-going ocular HSV infection, mAb 2c was systemically applied on day 6 post-infection. At this time point HSV-1 has already infected the contralateral retina (Vann and Atherton, 1991). Since the HSV-induced damage of the retina is completely irreversible (Francis et al., 2003), it was crucial to start the antibody treatment on day 6. Antibody-treated mice showed a decreased frequency of ARN (**Figure 1B**). After therapeutic mAb 2c treatment, 40% of the mice were protected from the development of disease, and 6/10 mice developed ARN (**Figure 1B**). Although the differences did not meet the level of significance, the data indicate that antibody-application under treatment-regiment may block the progression of retinal damage. Topical medication applied as eye drops or ointments are a convenient and safe treatment strategy for ocular infections (Bremond-Gignac et al., 2011). To determine whether the antiviral antibody could be effective in topical applications, mice were intracamerally infected and topically treated by periodical inoculations of the ipsilateral and contralateral eye with 5 μl (20 μg) mAb 2c containing eye drops starting at 24 h post-infection until day 12. Topical treatment showed almost no effect on the prevention of disease, since 80% (8/10) of the mice developed ARN (**Figure 1B**). Taken together, the antiviral treatment with mAb 2c or mAb hu2c revealed to be an effective option for the prevention of HSV-induced ocular disease. Notably, the early time point and the systemic route of application were crucial to mediate protection.

### Effect of Antibody-Based Immunotherapy on the Viral Loads in the Contralateral Eyes

We next investigated whether the antibody-based immunotherapy with mAb 2c and hu2c also affected the viral loads at the contralateral eyes. Therefore, mice (*n* = 6) were infected intracamerally and treated with the monoclonal antibodies as described above. PBS and ACV were used as controls. On day 8 post-infection, when the infection is expected to be manifested at the most regions of the contralateral retina (Vann and Atherton, 1991), the mice were sacrificed, the contralateral eyes collected, and stored at -80°C.

To determine the virus titres, eye specimens were homogenized at 4°C and analyzed by a standard plaque assay on Vero cells. High viral loads were detected in the contralateral eyes of the PBS-treated control mice (**Figures 2A,B**). Contralateral eyes from ACV-treated mice showed slightly decreased HSV-1 titres, but the difference did not reach the level of significance (**Figure 2A**). Consistent with the clinical findings, topical antibody treatment (only mAb 2c) had no significant impact on the viral loads (**Figure 2A**). Remarkably, systemically applied mAb 2c or mAb hu2c (P and PEP groups) significantly reduced the viral loads (**Figure 2A**). With one exception (mAb hu2c, prophylactic treatment), there was no virus detectable in the contralateral eyes (**Figure 2A**). Systemic antibody treatment starting at 6 days post-infection resulted in nearly unchanged viral loads when compared to PBS-treated control mice (**Figure 2B**). In agreement with the *in vivo* data, these results underline the importance of an early initiation of antiviral treatment for successful prevention of ARN.

### Biodistribution of mAb hu2c during the Initial HSV-1 KOS Infection in the Eye

Antibody-based immunotherapy with mAb 2c or hu2c mediated protection from the development of ARN. Furthermore, the viral loads at the contralateral eyes of antibody-treated mice were significantly reduced. The data indicate that the antibodies protect from disease by neutralizing virus or inhibiting the anterograde-directed virus spread. Virus neutralization commonly occurs at the site of infection (Koelle and Corey, 2003; Marasco and Sui, 2007). To clarify if the antibodies can enter the infected eyes, we next investigated whether the antibody can reach various compartments of the eye. To distinguish between the injected antibody and pre-existing antibodies, the biodistribution experiments were performed exclusively with the humanized antibody mAb hu2c. According to the well-established ARN mouse model (Vann and Atherton, 1991), only the contralateral eye was considered to estimate the antiviral efficacy of the antibodies in the previously described experiments. Since the manifestation of HSV-infection differs between the initially infected ipsilateral eye (iris, ciliary bodies) and the contralateral eye (retina), in the following experiments we examined the biodistribution of mAb hu2c in both eyes.

**FIGURE 3:**
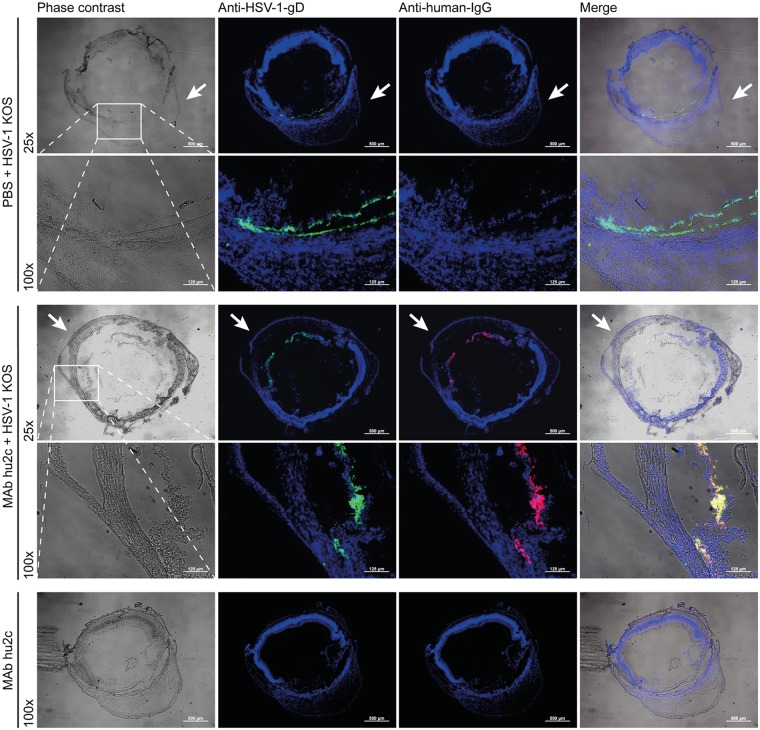
Distribution of mAb hu2c at the initially HSV-1-infected ipsilateral eye. Mice were infected by microinjection of 1 × 10^5^ PFU HSV-1 KOS into the anterior chamber of the right eye. MAb hu2c was intravenously applied at 48 h post-infection. Six hours after the antibody injection the ipsilateral eyes were removed. Naïve mice were accordingly treated with mAb hu2c as control. Cross-sections of these eyes were analyzed for HSV infection and the bound humanized antibody mAb hu2c. HSV-infected cells were stained with a mouse-anti-HSV-1/2-gD antibody and an Alexa488-conjugated goat-anti-mouse secondary antibody. MAb hu2c was detected with a Cy3-conjugated goat-anti-human-IgG secondary antibody. Magnification: 25× and 100× as indicated. Arrows indicate the cornea at the front section of the eye.

To induce the ARN, mice were infected by microinjection of HSV-1 KOS into the anterior eye chamber of the right eye. The humanized antibody mAb hu2c was intravenously injected at 48 h post-infection (initial infection) or on days 6, 7, and 8 after infection (contralateral infection of the retina). Six hours after injection of mAb hu2c the eyes were isolated, sectioned, and stained for fluorescence microscopy. Tissue sections of the eyes were examined for HSV-1 infection and bound humanized antibody. Uninfected eyes from mAb hu2c-treated mice served as control and showed no background staining (**Figure 3**).

Herpes simplex virus 1 infection could be detected in the area of the anterior chamber at the iris and ciliary body of the initially infected ipsilateral eye (**Figure 3**). Interestingly, bound humanized antibody was detected in the area of HSV-gD expression, demonstrating the ability of the antibody to diffuse into the eye and to bind specifically to HSV-infected tissues (**Figure 3**). No unspecific binding of mAb hu2c was detected in the ipsilateral eyes of uninfected mice (data not shown).

**FIGURE 4:**
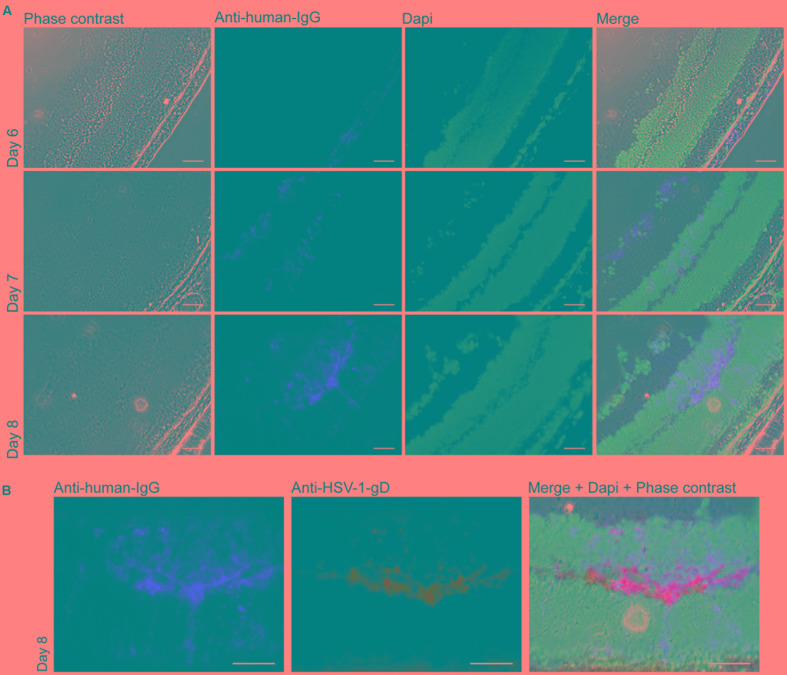
Distribution of mAb hu2c at the HSV-1-infected contralateral retina. **(A)** Mice were infected by microinjection of 1 × 10^5^ PFU HSV-1 KOS into the anterior chamber of the right eye. The HSV-gB-specific, humanized antiviral antibody mAb hu2c was intravenously applied on days 6, 7, or 8, the time window when the virus is expected to reach the contralateral retina (Vann and Atherton, 1991). Six hours after antibody injection, the eyes were removed and cross-sections of the contralateral retina were analyzed for mAb hu2c. Bound mAb hu2c was stained with a Cy3-conjugated goat-anti-human-IgG secondary antibody. **(B)** Additionally, cross-sections from eyes removed on day 8 were stained for HSV-1 infection with a mouse-anti-HSV-1/2-gD antibody and an Alexa488-conjugated goat-anti-mouse secondary antibody to verify co-localization of HSV-1 infection and bound mAb hu2c. Scale bars = 50 μM.

Bound mAb hu2c, as a marker for antibody distribution and HSV-1 infection, was detected on days 7 and 8 but not on day 6 after infection at the contralateral retina (**Figure 4A**). To verify these data, we additionally stained HSV-1-infected retinas from day 8 with a mouse anti-HSV-1/2-gD-antibody and an Alexa488-conjugated secondary antibody for HSV infection. The additional staining confirmed the co-localization of HSV-1 infection and bound mAb hu2c within infected retinal tissues (**Figure 4B**). These results provide for the first time evidence that mAb hu2c is able to enter the HSV-1-infected regions within the eyes.

### *In Vitro* Reactivation of HSV-1 from Trigeminal Ganglia of Mice Treated with mAb 2c or mAb hu2c

**FIGURE 5:**
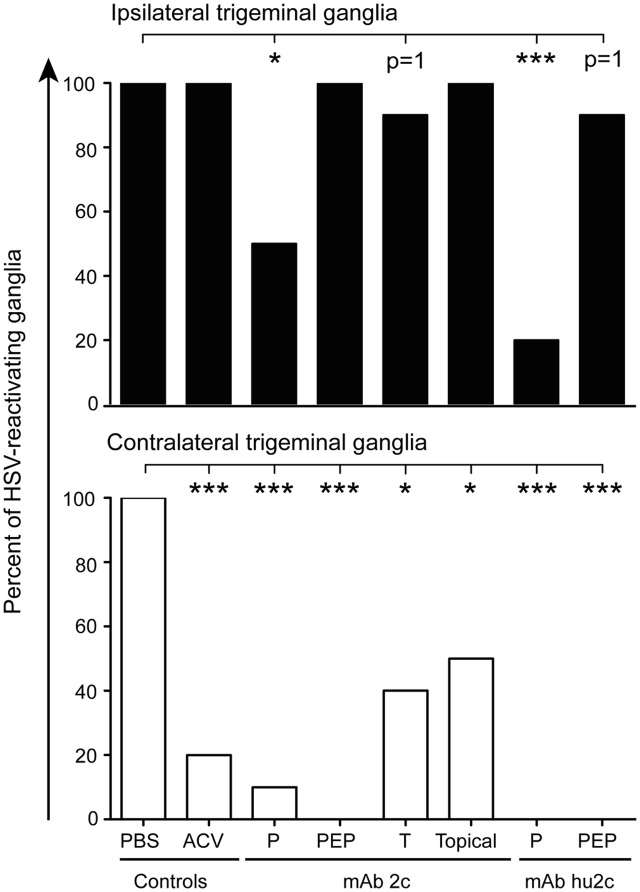
Reduced frequency of HSV reactivations from the trigeminal ganglia of antibody-treated mice. Mice (*n* = 10) were infected with HSV-1 KOS. The antibodies mAb 2c or mAb hu2c were applied at 24 h prior to infection (pre-exposure prophylaxis, P), at 24, 40, and 56 h after infection (PEP) or at day 6 post-infection (therapy, T, only mAb 2c). Topical treatment with mAb 2c was started at 24 h post-infection and performed five times per day by applying antibody containing eye drops until day 12. ACV standard therapy at 10 mg/kg body weight was performed three times a day by intraperitoneal injection. The percentages of reactivating ipsilateral or contralateral trigeminal ganglia from HSV-1 KOS-infected mice are shown. Trigeminal ganglia were isolated on day 12 after infection and co-cultured with Vero cells for 4 weeks. The occurrence of cytopathic effects was equated as reactivation. The differences in the total numbers of reactivating ganglia between the antibody treatment groups were compared with PBS and statistical significances were determined with the Fisher’s exact test. Comparisons were considered significant at ^∗^*P* < 0.05; ^∗∗^*P* < 0.01; and ^∗∗∗^*P* < 0.001.

The clinical ARN data imply that the antibodies mAb 2c and mAb hu2c might block the neuronal of HSV-1 transmission *in vivo*. To prove this hypothesis, we isolated the ipsilateral and contralateral TG from the HSV-1-infected and antibody or ACV-treated mice on day 12 post-infection and examined these ganglia for HSV reactivation *in vitro*. HSV reactivation was detected in all ipsilateral ganglia (10/10) isolated from PBS control mice or ACV-treated mice. Antibody treatment started at 24 h or later after HSV infection (PEP, therapy, topical treatment) had no impact on the frequency of reactivations from ipsilateral TG. In contrast, mice systemically treated with mAb 2c or mAb hu2c at 24 h before infection showed significantly lower reactivation rates when compared to the PBS control (**Figure 5**). Only 50% (mAb 2c) or 20% (mAb hu2c) of the ipsilateral ganglia were tested positive for HSV reactivation (**Figure 5**). At the contralateral side, HSV reactivation was detected in all PBS-treated control mice (10/10) (**Figure 5**). Strikingly, a significant reduction of HSV reactivating ganglia was found in all groups receiving antiviral treatment when compared to the PBS control (**Figure 5**). Viral recovery was detected in 20% of ACV-treated mice, 40% of mice receiving mAb 2c treatment on day 6 (therapy group), 50% of mice topically treated with mAb 2c containing eye drops, and only 10% of mice receiving mAb 2c prophylaxis at 24 h before infection (**Figure 5**). Importantly, no HSV reactivation was found in contralateral ganglia from mAb hu2c-treated mice receiving pre- or post-exposure prophylaxis or mAb 2c-treated mice receiving PEP (**Figure 5**). Taken together, these results indicate that the transmission of HSV-1 from the location of the primary infection (ipsilateral eye) to the ipsilateral or contralateral TG can be inhibited by mAb 2c and mAb hu2c.

### Inhibition of the Anterograde-Directed Transmission of HSV-1 from Neurons to Cells

**FIGURE 6:**
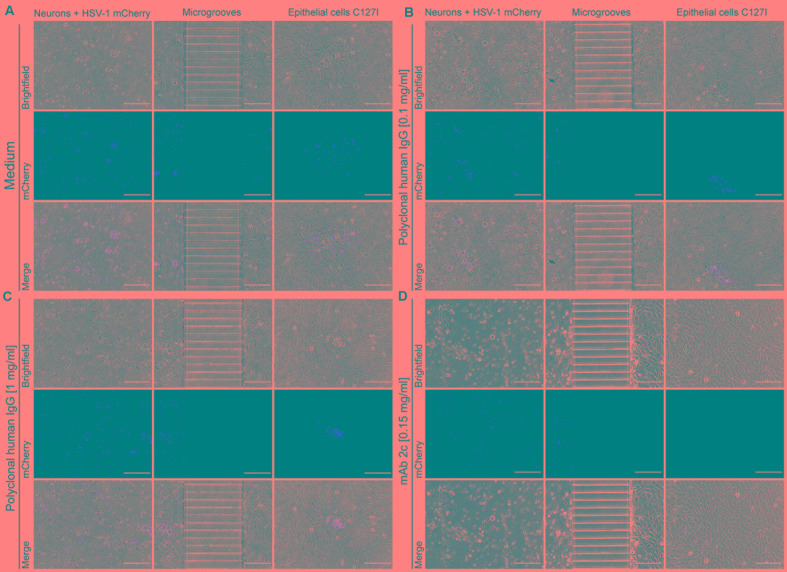
Inhibition of the anterograde-directed neuron-to-cell spread of HSV-1 by mAb 2c. The microfluidic chamber system (Taylor et al., 2005; Park et al., 2006; Liu et al., 2008) was used to analyze the impact of mAb 2c or polyclonal human antibodies on the axonal neuron-to-cell spread of HSV. Primary murine neurons were cultured for 14 days to allow the axons to spread from the somal side across the microgrooves to the axonal side. The neurons then were infected with an HSV-1(17^+^)Lox-CheVP26 reporter virus. Two hours after infection the inoculum was removed and C127I epithelial cells were seeded on the axonal side. Cells and neurons were then cultured for 48 h in the presence of cell culture medium **(A)**, polyclonal, HSV-neutralizing human IgG (Intratect^®^) **(B,C)** or mAb 2c **(D)** at the indicated concentrations. Magnification: 100×. Scale bar: 200 μM.

Next we aimed to better understand the underlying mechanism of protection mediated by mAb 2c or mAb hu2c in the mouse study. Based on the *in vivo* data, we hypothesized that mAb 2c, directed against the gB of HSV-1 and HSV-2, may directly inhibit the anterograde-directed spread of HSV-1 from neurons to epithelial cells. To investigate if mAb 2c can block this route of transmission *in vitro*, we used a microfluidic chamber system. This method is based on the co-culture of neurons and epithelial cells connected by axons in two fluidically isolated compartments (Taylor et al., 2005; Liu et al., 2008). Primary mouse neurons were cultured in the microfluidic chambers to allow the axons to pass through the microgrooves from the somal to the axonal compartment. The neurons then were infected with an HSV-1(17^+^)Lox-CheVP26 reporter virus. Two hours after infection the inoculum was removed and C127I epithelial cells were seeded on the axonal side. Thereby the cells attached and connected to the axons. The cells were cultured in the presence or absence of polyclonal human immunoglobulin as source of potent neutralizing antibodies or mAb 2c. Human polyclonal HSV-1 neutralizing antibodies were applied at concentrations up to 1 mg/ml. This value equates the antibody concentration estimated for the most human tissues (Shah and Betts, 2013). MAb 2c was applied at 150 μg/ml. After 48 h, the neurons had been efficiently infected with HSV-1 as indicated by mCherry expression in the initially infected neurons (**Figure 6**, somal compartments, left side). The antibody treatments had a different effect on the virus transmission from the neurons to the epithelial cells (**Figure 6**, axonal compartments, right side). Polyclonal human HSV-1 neutralizing antibodies showed no effect on the viral distribution across the microgrooves to the C127I epithelial cells (**Figures 6B,C**). There was no difference in plaque size between the cell cultures treated with polyclonal human immunoglobulin and medium control (**Figures 6A–C**). Remarkably, HSV-1 transmission was completely inhibited in the presence of mAb 2c (**Figure 6D**). These data demonstrate that mAb 2c, in contrast to polyclonal human immunoglobulin is capable of inhibiting the anterograde-directed spread of HSV from neurons to epithelial cells *in vitro*. Taken together, the results clearly support the hypothesis that mAb 2c and mAb hu2c are capable of inhibiting the anterograde-directed neuron-to-cell spread of HSV-1 and thereby mediate protection from the disease.

## Discussion

Herpes simplex viruses 1 and 2 are among the most ubiquitous human infections and persist lifelong in the host after primary infection (Whitley and Roizman, 2001). Recurrent ocular HSV infections are hard to remedy despite antiviral and anti-inflammatory treatment and thus are the major cause of infectious blindness in developed countries (Lau et al., 2007; Farooq and Shukla, 2012; Burrel et al., 2013). In this study we demonstrated that the HSV-1/2 gB-targeting antibody mAb 2c and its humanized counterpart mAb hu2c effectively protect mice from the development of severe ocular disease in a mouse model of ARN. Furthermore, we found that the anterograde-directed spread of HSV between neurons and epithelial cells can be completely inhibited by mAb 2c.

To determine the *in vivo* relevance of mAb 2c and hu2c in prevention and treatment of ocular HSV infections, we used a mouse model of ARN that mimics the anterograde spread of HSV and the onset of disease in humans (Whittum et al., 1984; Vann and Atherton, 1991). Systemically treated mice receiving antibody-based immunotherapy 24 h before or 24, 40, and 56 h after infection were completely protected from the development of ARN. A recognizable, but not significant protecting effect could be observed when the antiviral therapy was started at day 6 of infection, when the virus was expected to reach the contralateral cornea.

The route of application seems to be crucial to warrant proper distribution to the eye. Topically applied IgG-antibodies cannot reach deeper tissue sections of the eye, most probably due to their molecular size (Dastjerdi et al., 2011). In accordance with prior studies (Krawczyk et al., 2015), our experiments showed that systemic application is crucial for mediating protection from disease. These data indicate that the progression of disease could be limited by systemically administrating the antiviral antibodies.

Interestingly, the number of reactivating ganglia was significantly reduced in antibody- and ACV-treated mice. The neuronal spread of HSV also depends on the viral load (Mikloska et al., 1999). Therefore, a reduction of viral replication at the primary infected local tissues by neutralizing antibodies or aciclovir may contribute to suppress the viral transmission and explain the reduced numbers of reactivating TG. Another explanation may be that mAb 2c or hu2c directly interferes with the anterograde-directed transmission of HSV. To establish recurrent infection after reactivation, HSV migrates from the ganglia to the periphery. This anterograde-directed neuron-to-cell transmission includes the *trans*-synaptic spread of HSV between adjacent neurons and the axon-to-cell transmission resulting in the infection of peripheral tissues (Taylor et al., 2012b). The axon-to-cell transmission may occur by viral transmission through syncytial connections between neurons and epithelial cells or after egress of the virus from the axon and direct entry into the postsynaptic target cell after passing the intercellular gap (Mikloska et al., 1999; Smith et al., 2001; Curanovic and Enquist, 2009). Notably, a fusion step is required, either between the axon and the target cell or between the membrane of the egress virion and the target cell. The glycoprotein B has been described as the fusion protein of HSV and to be essential for virus entry and the intercellular spread (Heldwein et al., 2006; Sattentau, 2008). The gB-targeting monoclonal antibody mAb 2c recognizes a discontinuous, highly conserved HSV-1 and 2 epitope on HSV-gB and proofed to be highly effective in the prevention of genital HSV infections (Daumer et al., 2011; Krawczyk et al., 2011, 2013; Szpara et al., 2014).

We used the microfluidic chamber system to investigate whether monoclonal antibody mAb 2c may interfere with the intercellular transmission of HSV. This system serves as a model for the anterograde-directed spread of HSV infection from neurons to epithelial cells *in vitro* (Liu et al., 2008; Taylor et al., 2012a). We found that in contrast to polyclonal human HSV-1 neutralizing antibodies mAb 2c effectively blocks the neuron-to-cell transmission at a concentration of 1 μM (150 μg/ml). Polyclonal human antibodies applied at up to 1 mg/ml did not prevent on the viral transmission. The concentration of 1 mg/ml was chosen as it represents the average antibody concentration expected in most human tissues (Shah and Betts, 2013). At this concentration, human HSV neutralizing antibodies are not capable of inhibiting the neuron-to-cell spread. The remaining question is whether humans produce antibodies with cell-to-cell spread inhibiting properties. Prior studies tested antibodies that could only reduce but not inhibit the viral transmission between HSV-infected human fetal DRG neurons and autologous epidermal cells (skin explants) (Mikloska et al., 1999). The level of protection was directly correlated with the antibody concentration used in these experiments (Mikloska et al., 1999). Polyclonal human anti-HSV sera, a human monoclonal anti-gD antibody and polyclonal monospecific rabbit sera against HSV-1-gB or gD reduced the viral transmission from DRGs to epithelial cells (Mikloska et al., 1999). These findings suggest that human (and rabbit) antibodies might somehow interfere with the viral transmission via the cell-to-cell spread. The molecular mode of action of these antibodies is unclear. Since there was no internalization of the antibodies into neurons, the authors excluded that these antibodies inhibited viral replication or viral transport within the axons. Since these antibodies target the glycoproteins B and D, which are required for membrane fusion (Heldwein et al., 2006), it might be a fusion process that is blocked by these antibodies. Hence, the authors concluded that the inhibitory effects of polyclonal HSV-1-neutralizing sera or the anti-gD antibody on axonally transmitted HSV infection are mediated by targeting the virus passing across the intercellular gap, which requires a membrane fusion process (Mikloska et al., 1999).

Another study showed that the internalization of antibodies into neurons may occur primarily via clathrin-dependent Fcγ-receptor endocytosis (Congdon et al., 2013). Here, both the antibody and the neurons were of murine origin. This might be the prerequisite for antibody uptake. Therefore, internalization of antibodies (mAb 2c and primary neurons used in the present study were of murine origin) might also contribute to blocking of the viral assembly in the neurons or egress from the neurons. However, mAb 2c crosslinks gB and can protect cells from HSV infection (Krawczyk et al., 2011). Therefore, mAb 2c may interfere with the gB-mediated membrane fusion between the axonal terminal membrane and the target cell membrane. The block may also occur after virion egress from the axon by interfering with the viral entry into the postsynaptic epithelial cell. Thus, it seems more likely that mAb 2c perturbed the gB-driven fusion process required for infection. Interestingly, there was also a significantly lower number or reactivating ipsilateral ganglia isolated from prophylactically treated mice. Since a fusion processes also occur during the retrograde-directed *trans*-synaptic neuron-to-cell spread (Curanovic and Enquist, 2009), another possibility is that mAb 2c also can block this route of transmission. Further studies will clarify how far mAb 2c can also block the cell-to-neuron spread, a process that is essential for the establishment of latency in dorsal root ganglia (Whitley et al., 2007).

Taken together, the treatment of HSV-infected mice with the HSV-1/2 neutralizing, gB-specific antibody mAb 2c or its humanized counterpart mAb hu2c resulted in complete protection from the development of ocular herpes and the development of ARN. Furthermore, we demonstrated that the anterograde-directed neuron-to-cell spread of HSV can effectively be blocked by this antibody. Our work provides the basis for the humanized antibody mAb hu2c to become a powerful drug against ocular HSV infections.

## Author Contributions

DB and AK designed the study and wrote the manuscript. DB, MA, MD, AB, CH, UD, BG, AG, VP, MK, AE-H, BS, AH, MR, and AK performed the experiments and analyzed the data. All authors approved the final version of the manuscript.

## Conflict of Interest Statement

The authors declare that the research was conducted in the absence of any commercial or financial relationships that could be construed as a potential conflict of interest. The reviewer HF and handling Editor declared their shared affiliation.
